# Utility of the CAT in the therapy assessment of COPD exacerbations in China

**DOI:** 10.1186/1471-2466-14-42

**Published:** 2014-03-11

**Authors:** You-Hui Tu, Yan Zhang, Guang-He Fei

**Affiliations:** 1 Pulmonary Department, First Affiliated Hospital of Anhui Medical University, Hefei, 230022 Anhui, China

**Keywords:** Chronic obstructive pulmonary disease assessment test, Exacerbation, Inflammatory biomarkers, Pulmonary function, Therapy assessment

## Abstract

**Background:**

Chronic obstructive pulmonary disease (COPD) exacerbations are accompanied with increased systemic inflammation, which accelerate the pulmonary function injury and impair the quality of life. Prompt and effective treatments for COPD exacerbations slow down the disease progression, but an objective instrument to assess the efficacy of the treatments following COPD exacerbations is lacking nowadays. The COPD Assessment Test (CAT) is an 8-item questionnaire designed to assess and quantify health status and symptom burden in COPD patients. We hypothesize that the change in CAT score is related to the treatment response following COPD exacerbations.

**Methods:**

78 inpatients with clinician-diagnosed acute exacerbation of COPD (AECOPD) completed the CAT, St George’s Respiratory Questionnaire (SGRQ) and modified Medical Research Council (mMRC) Dyspnea Scale both at exacerbation and the 7th day of therapy, and a subgroup of 39 patients performed the pulmonary function test. Concentrations of serum C-reactive protein (CRP) and plasma fibrinogen were assayed at the same time. Correlations between the CAT and other measurements were examined.

**Results:**

After 7 days’ therapy, the CAT and SGRQ scores, mMRC grades, as well as the concentrations of CRP and fibrinogen all decreased significantly (P < 0.001). Meanwhile, the FEV1% predicted had a significant improvement (P < 0.001). The CAT scores were significantly correlated with concurrent concentrations of CRP and fibrinogen, SGRQ scores, FEV1% predicted and mMRC grades (P < 0.05). The change in CAT score was positively correlated with the change of CRP (r = 0.286, P < 0.05), SGRQ score (r = 0.725, P < 0.001) and mMRC grades (r = 0.593, P < 0.001), but not with fibrinogen (r = 0.137, P > 0.05) or FEV1% predicted (r = -0.101, P > 0.05). No relationship was found between the changes of SGRQ score and CRP and fibrinogen (P>0.05).

**Conclusions:**

The CAT is associate with the changes of systemic inflammation following COPD exacerbations. Moreover, the CAT is responsive to the treatments, similar to other measures such as SGRQ, mMRC dyspnea scale and pulmonary function. Therefore, the CAT is a potentially useful instrument to assess the efficacy of treatments following COPD exacerbations.

## Background

Chronic obstructive pulmonary disease (COPD) is a common chronic respiratory disease around the world and it has become an increasing public health concern to the Chinese medical community [1]. COPD is associated with periodic exacerbations which manifest as worsening of lung function and increased dyspnea, cough, and sputum production [2,3]. COPD exacerbations cause hospital admissions, morbidity and mortality, directly leading to the deterioration of health-related quality of life [4]. It’s generally believed that exacerbations are important targets for treatments and prevention of disease progression of COPD [2]. Clinicians routinely use various methods to assess the health status and response to treatments of COPD patients [5], but a simple and objective instrument to evaluate the treatments response following AECOPD is still lacking currently.

The COPD Assessment Test (CAT) is a recently introduced, patient-completed instrument to assess and quantify health-related quality of life and symptom burden in patients of COPD [6,7]. It comprises 8 questions, each presented as a semantic 6-point (0–5) differential scale, providing a total score out of 40. Scores of 0–10, 11–20, 21–30, 31–40 represent mild, moderate, severe or very severe clinical impact, respectively [8]. Evidence has shown its good internal consistency and test-retest reliability [7,9], and it is suitable for routine clinical use for both stable and exacerbating COPD [10-13]. Currently, the CAT has been translated into many languages and used worldwide, including China [14], but as yet no data is available on whether the CAT can be used to assess the response to treatments at exacerbations or therapeutic interventions in China.

COPD exacerbations usually accompanied with increased airway and systemic inflammation [15,16]. The dynamic changes of systemic inflammatory biomarkers following exacerbations can be used to evaluate the therapeutics efficacy [5]. An obvious symptom recovery and reduction in systemic inflammation can be seen in seven days following a positive treatment [15]. In this study, we hypothesize that the CAT scores following exacerbations are correlated with the systemic inflammation (evaluated by CRP and fibrinogen), mMRC grades, as well as the pulmonary function. Therefore, we propose that the CAT may be a potentially useful instrument to evaluate the therapeutic response to exacerbation treatments.

## Methods

### Subjects

**Figure 1:**
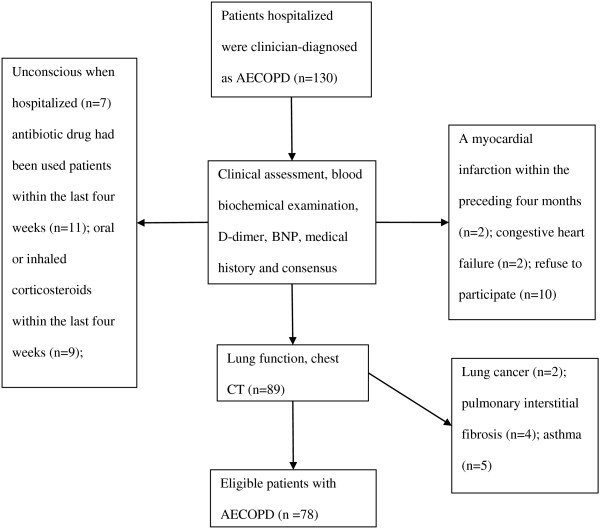
Strategies for screening patients with AECOPD.

The study involved patients with clinician-diagnosed COPD exacerbation, which defined as an acute event characterized by a worsening of respiratory symptoms (dyspnea, sputum purulence or sputum volume) that is beyond normal day-to-day variations and leads to a change in medication [2,17]. Subjects were recruited from the Pulmonary Department, First Affiliated Hospital of Anhui Medical University from August 2012 to January 2013. Patients with an established history of COPD were included, with a post-bronchodilator forced expiratory volume in one second (FEV1) to forced vital capacity (FVC) ratio <70% at diagnosis [2], in addition, they should be able to understand and complete the CAT, SGRQ and mMRC dyspnea scale independently. Exclusion criteria were as follows: Patients with a primary diagnosis of asthma, bronchiectasis or other active chronic respiratory disease requiring treatments, interventions or diagnostics, or any other severe or uncontrolled comorbidities; Patients with mental disorders or any other condition associated with an immuno-inflammatory response; Patients who had received antibiotic or corticosteroids during the past four weeks were also excluded. The study was approved by the ethics committee of First Affiliated Hospital of Anhui Medical University. All patients provided written consent to participate and were informed the possible risks of the study. Patients were screened according to the strategies illustrated in Figure 1.

Patients were recruited on the day they presented with an acute exacerbation. Demographic information, detailed medical and smoking history were record at recruitment. The exacerbation frequency, which was based on the number of exacerbations the patient recalled for the year prior to recruitment was also collected. Previous studies have shown a good correlation between exacerbation frequency recorded on diary cards and that remembered by the patient over the same one year period [18].

### Measurement of systemic inflammatory biomarkers and arterial blood gas analysis

Prior to treatments, peripheral venous blood samples of all subjects were taken to test the concentrations of serum CRP and plasma fibrinogen, which measured by the Modular Analytics E170 Module (Roche, Burgess Hill, UK) and the Claus method (IL ACL Top Coagulation Analyzer, Lexington, MA ,USA) respectively, the arterial oxygen (PaO2) and carbon dioxide tensions (PaCO2) were evaluated with a Stat Profile Critical Care Xpress (Nova Biomedical, Waltham, Mass, USA) while patients breathed room air in the supine position. After 7 days’ therapy, all subjects were required to take another venous blood sample to measure serum CRP and plasma fibrinogen following the methods mentioned above.

### Health-related quality of life questionnaires and mMRC dyspnea scale

Patients completed the CAT and SGRQ questionnaires and mMRC dyspnea scale independently at exacerbation and the 7th day of therapy. The effect on quality of life was measured by the CAT score.

### Pulmonary function test

Standardized pulmonary function test was performed both at exacerbation and the 7th day of therapy with a dry spirometer device (Erich Jaeger GmbH, Hoechberg, Germany) at 15 minutes after inhaling salbutamol 400 μg (Ventolin; GlaxoSmithKline; London, UK), and the FVC, FEV_1_, and FEV_1_/FVC ratio were recorded.

Patients at exacerbation were treated according to the prevailing guidelines and clinical judgment with inhaled short-acting β2-agonists, antibiotics, systemic corticosteroids and theophylline, etc. [2]. Neither the magnitude of the exacerbation CAT/SGRQ score nor the mMRC grades played any role in the treatment decisions. On the 7th day of therapy, the patients were required to have a statement on the global rating changes in COPD since the first day independently, with a six-point single-item classifying change as 'much worse’, 'worse’, 'no change’, 'better’, 'much better’, or 'completely resolved’ referred to the method Jones and his colleagues had used [12]. Responders were defined as having a global rating of change 'better’, 'much better’, or 'completely resolved’. Non-responders were defined as having a global rating of change 'no change’, 'worse’, or 'much worse’.

### Statistical analysis

Demographic and clinical characteristics of patients were summarized descriptively. Data were expressed as mean ± standard deviation. Skewed distribution data were made to normal distribution data by ㏒_10_ transformation. SPSS Statistics version 17.0 was used for statistical analysis. The Pearson regression was used to analyze the correlations between CAT scores and inflammatory biomarkers, SGRQ scores, FEV1% predicted, the correlations between the mMRC grades and CAT scores, inflammatory biomarkers were analyzed by analysis of variance (ANOVA). Group comparisons were tested using t-tests or Chi-Square test. A P value <0.05 was considered significant in all statistical analyses.

## Results

### Patient characteristics

A total of 130 AECOPD patients were screened and 78 patients were eligible. Their baseline characteristics are reported in Table 1, alongside the subgroup of 39 patients who also performed the pulmonary function test on the 7th day of therapy. No difference was found in their baseline characteristics (P > 0.05). After 7 days’ therapy, 76 patients were responders and only 2 patients were non-responders, the mean change in CAT score of the 78 patients was (-10.36 ± 5.03) units, among which the “responders” was (-10.49 ± 4.83) units and the “non-responders” was -0.93 units (the mean value of the two patients).

**Table 1:** Demographic characteristics of the total and subgroup patients

**Variable**	**Total group**	**Sub group**	**P value**
	**(n = 78)**	**(n = 39)**	
Age, years	69.01 ± 10.39	67.90 ± 11.02	0.56^a^
Females, n (%)	34(43.58)	18(46.15)	0.78^b^
BMI, kg/m^2^	21.82 ± 4.02	21.06 ± 3.87	0.89^a^
Smoking status, %			
Current smoker	35.14	40.93	0.66^b^
Ex-smoker	35.12	33.26	
Never smoked	29.74	25.81	
Smoking index, pack-years	43.00 ± 26.00	41.00 ± 23.00	0.67^a^
Comorbidities, n (%)	33(49.25)	18(46.15)	0.69^b^
Exacerbation frequency	2.55 ± 1.44	2.58 ± 1.62	0.92^a^
Education, years	6.79 ± 5.17	6.51 ± 4.89	0.63^a^
PaO2, mm Hg	67.05 ± 18.90	67.80 ± 23.40	0.54^a^
PaCO2, mm Hg	44.48 ± 11.85	43.42 ± 15.08	0.31^a^
Pulmonary function at exacerbation			
FEV1, % predicted	46.79 ± 19.14	45.37 ± 20.27	0.71^a^
FEV1, L	0.90 ± 0.33	0.82 ± 0.38	0.39^a^
FVC, L	1.57 ± 0.56	1.49 ± 0.57	0.44^a^

Data are presented as mean ± standard deviation, except where indicated otherwise. Comorbidities included hypertension, diabetes mellitus, cerebral infarction, coronary artery disease, and these comorbidities were all in stable state which confirmed by physicians from related departments. BMI = Body Mass Index. ^a^t-test. ^b^χ^2^ test.

### The relationships between the CAT, SGRQ, mMRC and inflammatory biomarkers

**Figure 2:**
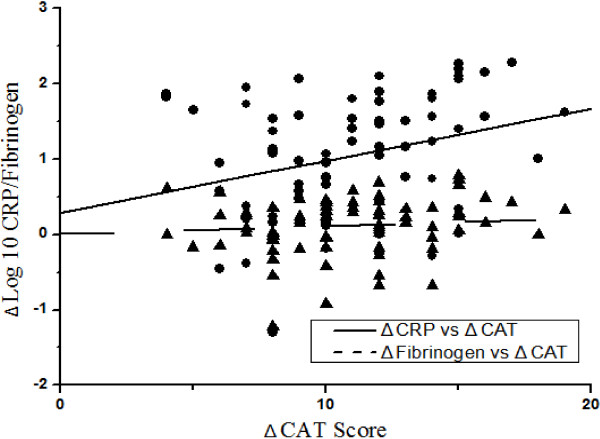
**Pearson correlations between the change in CAT score and the changes of inflammatory biomarkers.** ● solid line = Δ㏒_10_CRP vs ΔCAT: r = 0.286, P < 0.05; ▲ dash line = Δ㏒_10_ fibrinogen vs ΔCAT: r = 0.137, P > 0.05. Δ = the change of CAT scores and the concentrations of CRP and fibrinogen.

After ㏒_10_ transformation, the concentrations of CRP and fibrinogen were not affected by exacerbation frequency, comorbidities, or smoking status (P > 0.05). After 7 days’ therapy, the CAT and SGRQ scores, mMRC grades, as well as the concentrations of CRP and fibrinogen all had a significantly reduction (P < 0.001), as shown in Table 2. The CAT and SGRQ scores at exacerbation and the 7th day of therapy were all significantly related to concurrent levels of CRP and fibrinogen as shown in Table 3. The mean change in CAT score (-10.36 ± 5.03 units) following exacerbation was significantly related to the change in CRP (r = 0.286, P < 0.05), but not in fibrinogen (r = 0.137, P > 0.05), as shown in Figure 2. No relationship was found between the changes of SGRQ score (-13.47 ± 8.98 units) and CRP or fibrinogen (P > 0.05). Positive correlations were found between the mMRC grades and concurrent levels of CRP (exacerbation: r_s_ = 0.256, P < 0.05, the 7th day of therapy: r_s_ = 0.289, P = 0.01) and fibrinogen (exacerbation: r_s_ = 0.296, P < 0.01, the 7th day of therapy: r_s_ = 0.246, P < 0.05). The mean change of CRP (1.04 ± 0.79 mg/L) was positively related to that of fibrinogen (0.12 ± 0.38 g/L, r = 0.485, P < 0.001).

**Table 2:** CRP, fibrinogen, CAT and SGRQ scores, mMRC grades before and after therapy (n = 78)

	**Exacerbation**	**The 7th day**	**P value**
㏒_10_CRP, mg/ L	1.14 ± 0.69	0.39 ± 0.52	<0.001
㏒_10_fibrinogen, g/ L	0.59 ± 0.16	0.37 ± 0.16	<0.001
SGRQ	55.17 ± 17.07	41.69 ± 16.48	<0.001
CAT	23.19 ± 7.00	12.83 ± 5.82	<0.001
mMRC grades	2.73 ± 0.99	1.36 ± 0.91	<0.001

Data are presented as mean ± standard deviation, P value was calculated by paired t-test.

CRP = C-reactive protein, CAT = COPD Assessment Test, SGRQ = St George’s Respiratory Questionnaire.

**Table 3:** Relationships between CAT, SGRQ scores and CRP, fibrinogen (n = 78)

	**Exacerbation**		**The 7th day**	
	**r**	**P value**	**r**	**P value**
CAT vs ㏒_10_CRP	0.398	<0.001	0.409	<0.001
CAT vs ㏒_10_fibrinogen	0.392	<0.001	0.262	<0.05
SGRQ vs ㏒_10_CRP	0.282	<0.05	0.354	0.001
SGRQ vs ㏒_10_fibrinogen	0.292	0.01	0.297	<0.01

CRP = C-reactive protein, CAT = COPD Assessment Test, SGRQ = St George’s Respiratory Questionnaire. r = Pearson Correlation coefficient.

### The CAT and exacerbation frequency

The mean CAT scores of the 78 patients at exacerbation and the 7th day after of therapy were 23.19 ± 7.00 and 12.83 ± 5.82, respectively, Patients with frequent exacerbations (≥2 exacerbations during the past year, n = 60) had a significantly higher mean exacerbation CAT score of 24.74 ± 7.21 compared to infrequent exacerbations (<2 exacerbations during the past year, n = 18) with a mean exacerbation CAT score of 20.62 ± 5.75 (P < 0.001). After 7 days’ therapy, frequent exacerbators still had a higher CAT score of 15.35 ± 6.00 than infrequent exacerbators with a CAT score of 10.62 ± 4.66 (P < 0.005). The mean exacerbation frequency was 2.55 ± 1.44 times during the past one year.

### The CAT and SGRQ, mMRC

**Figure 3:**
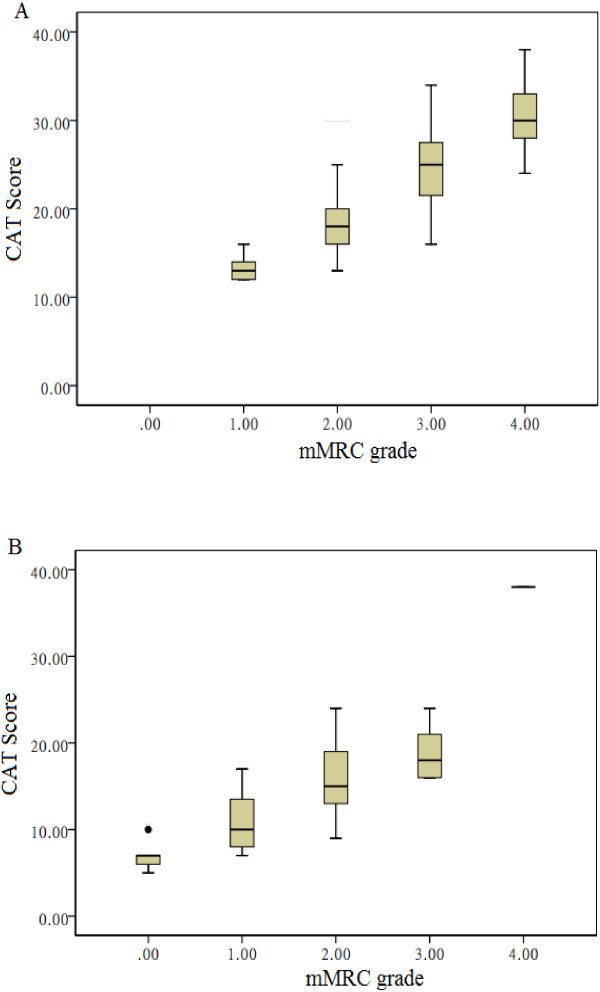
**Box plots of CAT scores in patients grouped mMRC grades at exacerbation (Figure**3**A) and the 7th day of therapy (Figure**3**B).** The horizontal line through each box represents the median. The ends of each box represent the 25th and 75th percentile locations, and the lines represent the range of the date. The cross of each box represents the mean.

The CAT scores were positively correlated with concurrent SGRQ scores (exacerbation r = 0.831, the 7th day of therapy r = 0.774, both P < 0.001) and mMRC grades (exacerbation r_s_ = 0.832, the 7th day of therapy r_s_ = 0.786, both P < 0.001). The mean change in CAT score was also significantly related to that of SGRQ score (r = 0.725, P < 0.001) and mMRC grades (r_s_ = 0.593, P < 0.001). The effect on quality of life according to the CAT scores and the mMRC grades of the total subjects was shown in Table 4. Figure 3 showed the distribution of the CAT scores grouped by the mMRC grades at exacerbation and the 7th day of therapy.

**Table 4:** CAT stages and mMRC grades before and after therapy (n = 78)

	**Exacerbation**	**The 7th day**	**P value**
	**n (%)**	**n (%)**	
CAT score			<0.001
0-10	0(0.00)	33(42.31)	
11-20	31(39.75)	36(46.15)	
21-30	35(44.87)	8(10.26)	
31-40	12(15.38)	1(1.28)	
mMRC grades			<0.001
0	0(0.00)	15(19.23)	
1	9(11.54)	27(34.62)	
2	24(30.77)	30(38.46)	
3	24(30.77)	5(6.41)	
4	21(26.92)	1(1.28)	

P value was calculated byχ^2^ test.

### The CAT and pulmonary function

**Figure 4:**
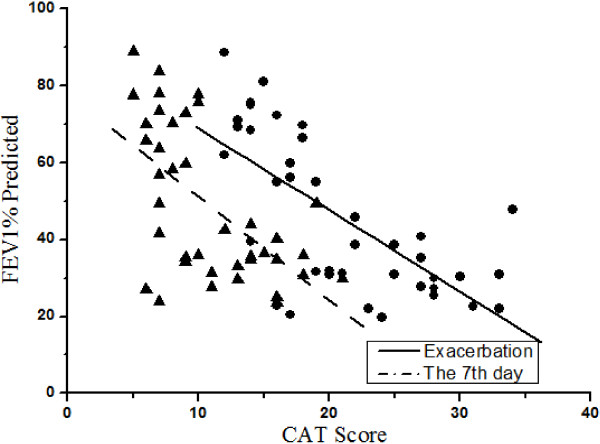
**Pearson correlations between CAT scores and FEV1% predicted.** ● solid line = Exacerbation: r = -0.683; ▲ dash line = the 7th day after therapy: r = -0.591, both P < 0.001.

A subgroup of 39 patients performed the pulmonary function test both at exacerbation and the 7th day of therapy. Significant improvement was found in FEV1% predicted on the 7th day of therapy (P < 0.001), as the mean value at exacerbation and the 7th day after therapy was (45.37 ± 20.27) % and (48.80 ± 19.84) %, respectively. Significantly negative correlations were found between the CAT scores and contemporaneous FEV1% predicted (exacerbation r = -0.683, the 7th day after therapy r = -0.591, both P < 0.001, Figure 4), but no correlation was found between the changes of CAT score and FEV1% predicted (r = -0.101, P > 0.05).

No difference in CAT score was found between smokers and non-smokers, as well as in patients with comorbidities or without comorbidities (P>0.05).

## Discussion

This study, to our knowledge, is the first time to use the CAT to assess the treatment response and health status following exacerbations of COPD in China. Results have shown that the CAT and SGRQ scores, mMRC grades and the concentrations of CRP and fibrinogen during exacerbation all decreased significantly after 7 days’ therapy. Meanwhile, the FEV1% predicted had a significant improvement. The CAT scores were significantly correlated with concurrent levels of CRP and fibrinogen, mMRC grades, SGRQ scores and FEV1% predicted. The change in CAT score was related to that of CRP, SGRQ score and mMRC grades. Furthermore, we have shown that the CAT scores were significantly higher in patients with a history of frequent exacerbations.

COPD is associated with increased systemic inflammation [19,20], and the inflammation is aggravated during exacerbations [15,16]. Recent advances using inflammatory biomarkers suggest that quantification of serum proteins are effective in the diagnosis, classification, prognosis, and treatment response of COPD [5,21]. Both CRP and fibrinogen are acute-phase inflammatory biomarkers that are normally used in clinic, their concentrations will generally rise in response to infections but decline with the clinical recovery [22,23]. In this study, we found most patients had much higher concentrations of CRP and fibrinogen at exacerbation, probably triggered by bacterial and/or viral infections [24,25]. With the symptoms recovered, both CRP and fibrinogen had a significant reduction corresponding to the decrease of systemic inflammation, reflecting an effective treatment during the seven days. Our results showed that moderate but significan correlations (r = 0.262 to 0.409) were found between the levels of CRP and fibrinogen and concurrent CAT scores, SGRQ scores, and mMRC grades, these are reasonable as the clinical symptoms and poor health status in COPD patients are caused by a great many factors including the systemic inflammation [2,26]. Meanwhile, the inflammatory response may lag behind clinical and biochemical response following AECOPD [27], which can partly accounts for the weak correlations between inflammatory mediators and CAT/SGRQ scores. No relationship was found between the changes of SGRQ scores and CRP or fibrinogen after 7 days’ therapy. We speculate this attributes to the relatively weaker correlations between the SGRQ scores and CRP and fibrinogen following exacerbations as compared with the CAT scores, thus suggesting the CAT is more sensitive to the inflammatory response following exacerbations. More studies, probably of longitudinal nature will be required to test and verify these associations.

With the simplicity and reliability of CAT, it is now recommended to assess the symptom burden and health status of COPD patients [2]. The SGRQ is a disease-specific measure of health status for COPD. In the present study, significantly positive correlations were found between the CAT and SGRQ scores both at exacerbation and the 7th day of therapy, which was consistent with the initial validated studies [7]. Previous studies have shown that the relationship between the CAT and SGRQ is constant across the scaling range [28], and the mathematical relationship is CAT = 0.4 × SGRQ. However, what is interesting in our study is that the CAT score (12.83 ± 5.82 units) seems very small compared to SGRQ (41.69 ± 16.48 units) on the 7th day of therapy. We believe that the difference may be caused by the higher sensitivity of CAT to the clinical response of treatments following exacerbations than that of SGRQ. Mackay et al. have demonstrated that the CAT can provide a score of COPD exacerbation severity and model recovery following exacerbations [13]. Furthermore, the CAT is responsive to pulmonary rehabilitation and can distinguish different levels of response [29,30]. Our study complements the existing work by demonstrating that the CAT is an objective measurement to assess the treatment response following COPD exacerbations. This has particular relevance as the CAT provides an additional tool to assess the ability of novel interventions to reduce exacerbation severity and make comparisons across studies easier. However, we found that the mean change in CAT score over the 7 days in our study was quite dramatic (-10.36 ± 5.03 units) compared to previous studies [12,13]. We speculate there are two main factors, one is that most patients in the present study came from the countryside and they received less formal treatment in stable state, which made them are more sensitive and effective to the treatments when compared with those who had received the guidelines recommended treatments; Meanwhile, these patients were in low economic state and had more serious symptoms when hospitalized at exacerbations of COPD.

Dyspnea is the most prominent and distressing symptom of COPD patients. With the disease progresses, the severity of dyspnea and the frequency of exacerbations also increased, leading to a reduction in patients’ daily activities and health-related quality of life. In the present study, the CAT scores were positively correlated with contemporaneous mMRC grades, which was consistent with studies conducted by Zhou and Jones [31,32]. Both CAT and mMRC Dyspnea Scale were recommended to assess the symptom burden of COPD patients in the revised global strategy [2]. Considering dyspnea is not the only symptom that affects health status, and the CAT has a broader coverage of the impact of COPD on the patient’s daily life and well-being. Therefore, it is more appropriate to use the CAT as a symptom burden and heath status measurement. COPD patients are accompanied with persistent airflow limitation. Exacerbations can accelerate the decline of lung function and often with an incomplete symptomatic or physiological resolution [33], thus leading to reduced physical activities and poorer quality of life. In our study, the CAT scores were significantly and modestly related to contemporaneous lung function impairment (measured by FEV1% predicted), in accordance with previous data [13]. However, we found that the change in CAT score was not related to that of FEV1% predicted, probably due to the recovery of physiological function lags behind the symptoms recovery. Exacerbation frequency is associated with the health status impairment of COPD patients [34]. Our study demonstrated that frequent exacerbators have higher CAT scores than infrequent exacerbators during exacerbations, which is expected because the CAT scores in stable COPD patients with a history of frequent exacerbations are significantly higher than infrequent ones [13].

There are some limitations in our study. Firstly, the information in stable state of the subjects (basal CAT and SGRQ scores, pulmonary function parameters and mMRC grades, etc.) were absent, thus lacking the comparisons between stable state and exacerbation and recovery. Secondly, this study has the relatively small size of the participant population, especially the subjects who performed the spirometry on the 7th day of therapy. The small sample size may leads to the weak correlations between some measurements in our results, as well as some mild difference between our results and previous studies.

## Conclusions

In conclusion, the CAT is associate with the changes of systemic inflammation following exacerbations, and it is as responsive to the treatments as other measures such as SGRQ, mMRC dyspnea scale and pulmonary function. Thus the CAT scores can reflect the health status of COPD patients, and are potentially useful to assess the treatments response following COPD exacerbations. In addition, the CAT is particular useful for health settings where access to other objective measurements is limited.

## Competing interests

None of the authors has a financial relationship with a commercial entity that has an interest in the subject of this manuscript.

## Authors’ contributions

All authors participated in the conception and design of experiment. Y-HT carried out the experiment and draft the manuscript. The statistical analysis was performed by Y-HT and YZ. The manuscript was revised coordination and critically by G-HF. All authors read and approved the final manuscript.

## Pre-publication history

The pre-publication history for this paper can be accessed here:

http://www.biomedcentral.com/1471-2466/14/42/prepub
